# Characterization of a Novel Glucokinase Activator in Rat and Mouse Models

**DOI:** 10.1371/journal.pone.0088431

**Published:** 2014-02-12

**Authors:** Min Lu, Pingping Li, Gautam Bandyopadhyay, William Lagakos, Walter E. DeWolf, Taylor Alford, Mark Joseph Chicarelli, Lance Williams, Deborah A. Anderson, Brian R. Baer, Maralee McVean, Marion Conn, Murielle M. Véniant, Peter Coward

**Affiliations:** 1 Department of Medicine, University of California San Diego, La Jolla, California, United States of America; 2 Array BioPharma Inc., Boulder, Colorado, United States of America; 3 Amgen Inc., South San Francisco, California, United States of America; 4 Amgen Inc., Thousand Oaks, California, United States of America; INRA, France

## Abstract

Glucokinase (GK) is a hexokinase isozyme that catalyzes the phosphorylation of glucose to glucose-6-phosphate. Glucokinase activators are being investigated as potential diabetes therapies because of their effects on hepatic glucose output and/or insulin secretion. Here, we have examined the efficacy and mechanisms of action of a novel glucokinase activator, GKA23. In vitro, GKA23 increased the affinity of rat and mouse glucokinase for glucose, and increased glucose uptake in primary rat hepatocytes. In vivo, GKA23 treatment improved glucose homeostasis in rats by enhancing beta cell insulin secretion and suppressing hepatic glucose production. Sub-chronic GKA23 treatment of mice fed a high-fat diet resulted in improved glucose homeostasis and lipid profile.

## Introduction

Type 2 diabetes is preceded by insulin resistance, the impaired ability of tissues to take up and store glucose in response to insulin [1]. To compensate for this reduced responsiveness, the pancreatic β cells over-secrete insulin, causing hyperinsulinemia. Over time, the β cells lose their ability to compensate and “burn out”, at which point insulin levels fall irrespective of prevailing glucose levels [2], leading to hyperglycemia. Current treatment options for type 2 diabetes can be categorized into three main classes: biguanides, which mainly inhibit gluconeogenesis; insulin sensitizers, which promote insulin action; and insulin secretagogues or insulin itself, which raise insulin levels [3]. Refractory cases of type 2 diabetes typically require combinatorial therapy [4], and thus developing effective and safe treatments that target new pathways is important to help patients achieve optimal plasma glucose levels.

Glucokinase (GK) is an enzyme that catalyzes the phosphorylation of glucose to glucose-6-phosphate in the presence of ATP [5]. Glucose enters cells via specific transporters, but once inside it can freely diffuse out unless it is phosphorylated by glucokinase or other hexokinases [6]. This phosphorylation event helps to keep glucose inside the cell and is the first step of both glycogen synthesis and glycolysis [7]. Unlike other hexokinases, which are ubiquitously expressed in most cell types, GK is predominantly expressed in the liver, pancreas, intestine, and brain of rodents and humans [5]. In addition, compared to other hexokinases, GK has a lower affinity for glucose, displays substrate cooperativity, and is not inhibited by its product, glucose-6-phophate [5]. These characteristics allow GK to serve as a glucose sensor to regulate cellular metabolism in response to changes in glucose concentration [7]. For instance, in β cells, GK is the rate-limiting enzyme in glucose metabolism and thereby controls glucose-stimulated insulin secretion. In the liver, GK activation reduces hepatic glucose output or enhances glucose uptake due to its restraining effect on intracellular free glucose. Human genetic mutations in GK further underscore its role in controlling metabolic homeostasis: activating mutations result in hyperinsulinemia and hypoglycemia, and loss-of-function mutations result in a form of diabetes known as maturity onset diabetes of the young, type 2 (MODY2) [8].

The pharmaceutical industry has synthesized multiple small molecule compounds that bind to GK at a site distinct from the glucose-binding site and act to increase GK activity [7]. These glucokinase activators, or GKAs, have been shown to improve glucose homeostasis in preclinical models and in human diabetic patients [9], [10]. However, GKA treatment has also been associated with undesirable effects. As with insulin and insulin secretagogue therapies, patients treated with GKAs may experience hypoglycemic episodes due to over-secretion of insulin [11]. In addition, recent clinical trials have revealed increased circulating triglyceride (TG) levels [11], [12] and blood pressure [11] in patients taking GKAs. Also, unexpectedly, the efficacy of these GKA appeared to decrease over time, suggesting a potential lack of durable response [11], [12].

In the current study, we characterized a novel GKA, GKA23, in vitro and in preclinical rodent models to help further understand the mechanism of action of this class of compounds. We show that in rats GKA23 improved glucose homeostasis due to increased β cell insulin secretion and decreased hepatic glucose production. Sub-chronic treatment with GKA23 in a diet induced obesity (DIO) mouse model exhibited desirable durability and improved both glucose homeostasis and lipid profile.

## Materials and Methods

### Chemicals

(S)-1-(5-(3-(2,6-dimethylpyridin-3-yloxy)-5-(pyridin-2-ylthio)pyridin-2-ylamino)-1,2,4-thiadiazol-3-yl)ethane-1,2-diol hydrochloride (GKA23) was synthesized at Array BioPharma via the methods of Aicher et. al. [WO 2009042435] from commercially available starting materials. Captisol was purchased from Cydex (now Ligand Pharmaceuticals, Inc.). All other chemicals were purchased from Sigma-Aldrich.

### Enzyme activity assays

Glucokinase activity was measured using a kinetic, coupled reaction in which the glucose-6-phosphate generated by glucokinase is used as the substrate for glucose-6-phosphate dehydrogenase to generate 6-phosphoglucono-δ-lactone. The latter reaction converts NAD^+^ to NADH which is measured by absorbance at 340 nm. EC_50_ values were determined using recombinant rat or mouse glucokinase in the presence of 50 mM K^+^MOPS, pH 7.2, 250 mM KCl, 10 mM MgCl_2_, 1 mM DTT, 0.05% Triton X-100, 2% DMSO, 1 mM ATP, 1 mM NAD^+^, and 5 U/mL *Leuconostoc mesenteroides* glucose 6-phosphate dehydrogenase, and 5 mM glucose. *S*_0.5_ and maximal velocity were determined in the same buffer with glucose ranging over a 10-point dose-response curve beginning with a top dose of 80 mM and GKA23 ranging over an 8-point dose response beginning at a top dose of roughly 25 times the EC_50_.

### In vitro glucose uptake assay

Primary hepatocytes from 12 week old male Sprague Dawley rats (Invitrogen) were plated on collagen-coated Cytostar-T 96 well plates at a concentration of 30,000 cells/well and allowed to attach for 4 hours. The media was then replaced with Williams' E medium containing 5% FBS, 1 µM dexamethasone, and 4 µg/mL human insulin. After overnight incubation, the media was changed to Williams' E medium with 100 ng/ml insulin. After an additional 42–48 hours, the cells were incubated for 30 minutes in DMEM without phenol red and containing 2 mM glucose, 10 nM dexamethasone, and 0.1% BSA. Media was then replaced with DMEM without phenol red and containing 5 mM glucose, 10 nM dexamethasone, 0.1% BSA, and 5.6 µCi/mL 2-deoxy-D-[^3^H]-glucose and the cells incubated for 2 hours in the presence of GKA23. The cells were then washed with cold PBS containing 20 mM glucose and the plate read on a Microbeta reader.

### Animals

Male Sprague Dawley (SD) rats at 7 weeks of age were obtained from Charles River Laboratories (Wilmington, MA). Male wild type C57BL6 mice at 8 weeks of age were obtained from The Jackson Laboratory. Animals were housed on a 12 h: 12 h light: dark cycle with the lights on at 0600 h and were fed ad libitum except during the experiments.

Rat and mouse procedures conformed to the Guide for Care and Use of Laboratory Animals of the US National Institutes of Health, and were approved by the Animal Subjects Committee of the University of California, San Diego, CA, USA.

### Metabolic studies

Glucose and pyruvate tolerance tests, and all clamp studies were performed with 6 hour fasted animals, and GKA23 was administered by intraperitoneal (i.p.) injection at 30 mg/kg in 10% Captisol in water, unless indicated otherwise. For glucose tolerance tests, animals were injected i.p. with either vehicle (10% Captisol in water) or GKA23 30 min before the oral gavage with glucose (1 g/kg) or i.p. injection with pyruvate (1 g/kg).

For the hyperglycemic step clamp study, rats were fasted for 5.5 hours. Cannulated rats were intravenously injected with a priming dose of either vehicle or GKA23 (10 mg/kg), followed by a constant infusion of GKA23 in 10% Captisol at a rate of 5.5 mg/kg/hour. Thirty minutes later, blood samples were taken from the cannulated carotid artery at the basal state and glucose was then infused at a low rate to achieve a 20 min 120 mg/dl plateau within 1.5 hours. When blood glucose levels stabilized, blood samples were taken for measurement of insulin and C-peptide. Next, the glucose infusion rate was increased to maintain a 20 min steady state of 180 mg/dl, followed by the last step at 240 mg/dl. Blood samples were again taken for insulin and C-peptide measurement.

Rat hyperinsulinemic euglycemic clamp studies were performed as previously described [13]. Briefly, dual jugular venous cannulae and one carotid arterial cannula were implanted in rats. After 4 to 5 days of recovery, the hyperinsulinemic euglycemic clamp experiments began with a priming injection (7.5 µCi/0.2 ml) and constant infusion (0.25 µCi/min) of D-[3-^3^H] glucose (Du Pont-NEN, Boston, MA). Vehicle (10% Captisol in water) or GKA23 (30 mg/kg) was injected i.p. at −30 min. After 60 min of tracer equilibration and basal sampling at t = −10 and 0 min, glucose (50% dextrose, variable infusion; Abbott) and tracer (0.25 µCi/min) plus insulin (4 mU/kg/min) were infused into the jugular vein. The achievement of steady-state conditions (100 mg/dl±5 mg/dl) was confirmed at the end of the clamp by measuring blood glucose every 10 min and ensuring that steady state for glucose infusion and plasma glucose levels were maintained for a minimum of 30 min. Blood samples were taken at t = −10, 0 (basal), 110, and 120 (end of experiment) min to determine glucose-specific activity, insulin and free fatty acid (FFA) levels. All blood samples were immediately centrifuged, and plasma was stored at −80°C for subsequent analysis.

For the hypoglycemic clamp study, cannulated rats were treated with vehicle or GKA23 thirty minutes before the start of insulin infusion (2.5 mU/kg/min). Glucose infusion was also started to adjust blood glucose at 60 mg/dl at the 2 hour time point. Blood samples were taken for C peptide measurement.

To assess the effect of GKA23 in prolong fasted animals, rats were fasted for 24 hours followed by injection with either vehicle or GKA23. Blood samples were taken from tail vein at 5, 10, 30, 60, and 120 min after i.p. injection.

Glucose, insulin and FFA levels were measured as previously described [13].

### Long term treatment studies

For the long term diet induced obesity study, 12 week old C57BL6N mice were fed with 60% HFD (Research Diets, New Brunswick, NJ) for 10 weeks. Animals were injected i.p. daily with GKA23 or vehicle and, after 12 days, oral glucose tolerance tests were performed thirty minutes after compound or vehicle administration. Serum and hepatic lipid measurements were made on day 14. Food intake and body weight were monitored daily.

### Statistical analysis

Unless otherwise noted, data were analyzed by ANOVA followed by Tukey post hoc tests. Individual pair-wise comparisons were performed using Student's t test. Analysis was performed using Excel (Microsoft, Redmond, WA) or Prizm (GraphPad Software Inc., San Diego, CA).

## Results

**Figure 1:**
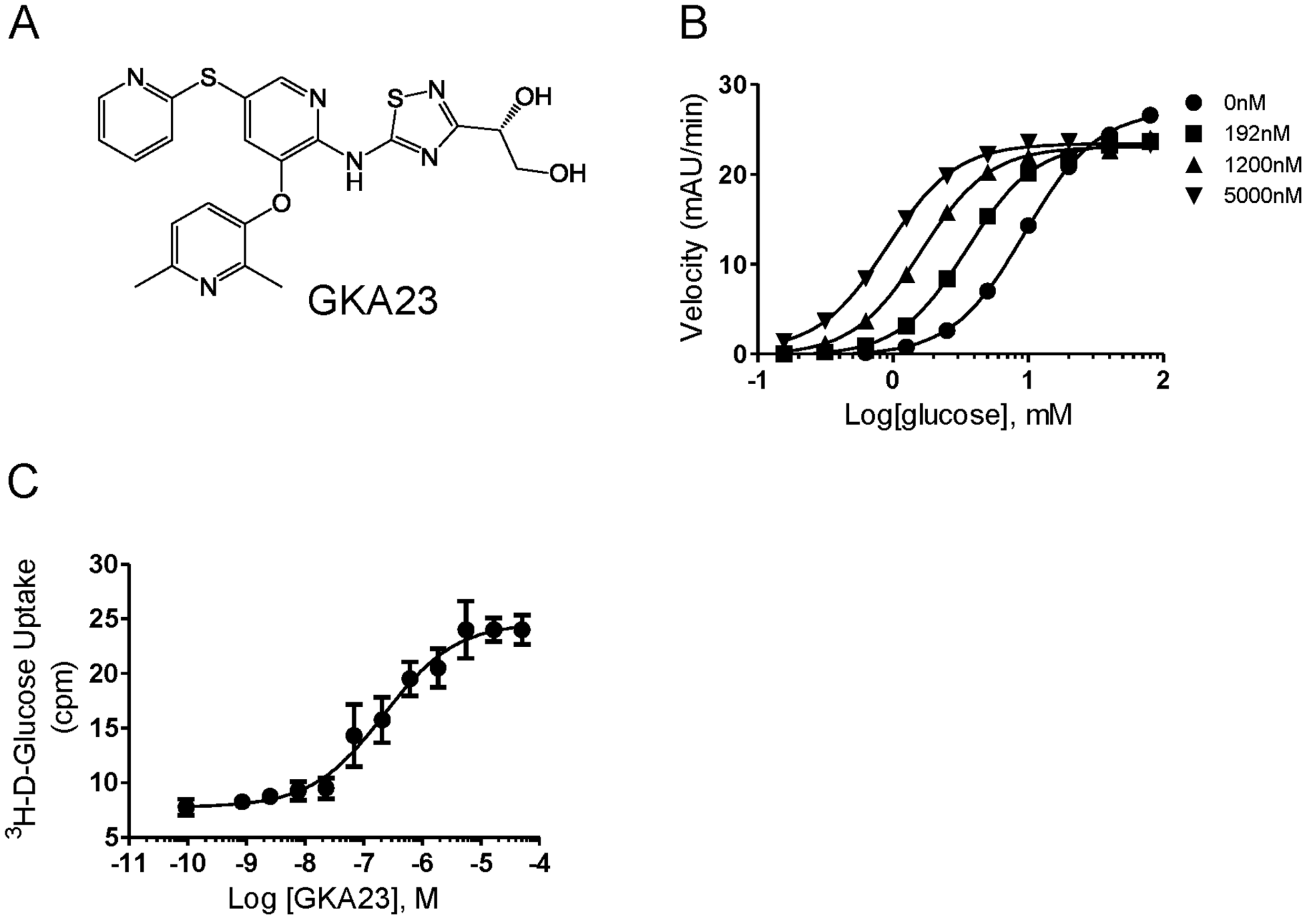
Structure and in vitro characterization of GKA23. (A) The chemical structure of GKA23. (B) Kinetic analysis of rat GK in the presence of increasing concentrations of GKA23. The *S*_0.5_ was 0.54±0.05 mM (n = 5). (C) ^3^H-deoxy glucose uptake in primary rat hepatocytes. The EC_50_ was 201±149 nM (n = 4).

The structure of GKA23 is shown in Figure 1A. In a biochemical assay using recombinant rat glucokinase, GKA23 increased the affinity of the enzyme for glucose by 18-fold (*S*_0.5_ = 0.54±0.05 mM vs 9.59±0.19 mM in the absence of activator) and reduced the maximal velocity by 15±3% (n = 5) (Fig. 1B). The EC_50_ for glucokinase activation in 5 mM glucose was 152±2 nM (n = 2). When tested with mouse glucokinase, GKA23 increased the affinity for glucose by 17-fold (*S*_0.5_ = 0.67±0.1 mM vs 11.31±0.38 mM in the absence of activator) and the maximum velocity by 24±8% (n = 5). The EC_50_ with 5 mM glucose was 267±97 nM (n = 2). As expected, GKA23 increased glucose uptake in primary rat hepatocytes (EC_50_ = 201±149 nM (n = 4), Fig. 1C).

**Figure 2:**
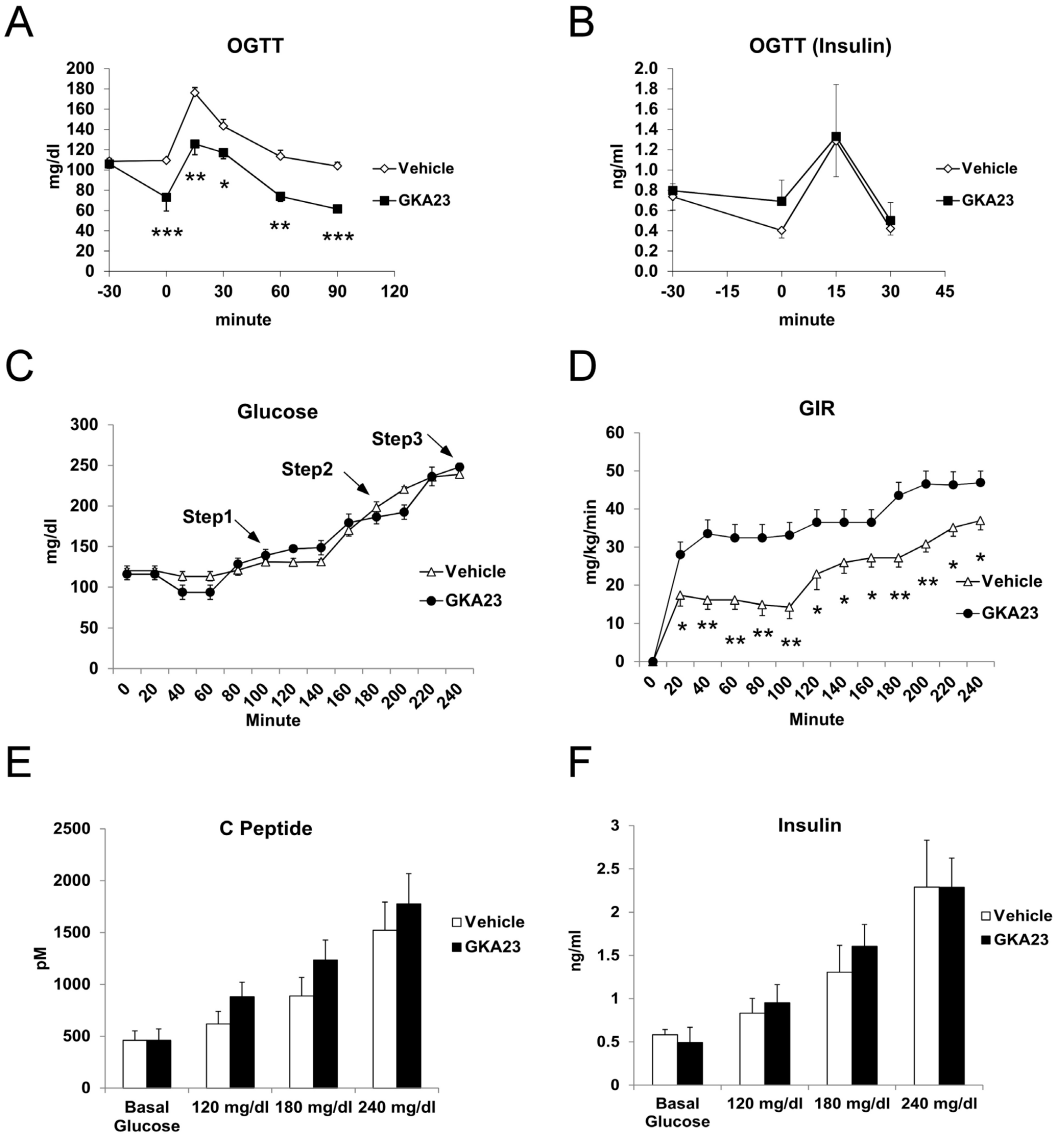
GKA23 improves glucose metabolism in rats during euglycemic and hyperglycemic state. (A) Oral glucose tolerance tests (OGTT; 1 g/kg) with 6 hour-fasted rats (n = 6/group). Vehicle or GKA23 (30 mg/kg) were administered i.p. at −30 min and glucose was orally gavaged at 0 min. (B) Plasma insulin concentration during OGTTs in 6 hour-fasted rats (n = 6/group). (C) Hyperglycemic step clamp study on control or GKA23 treated rats (n = 6/group) showing plasma glucose levels measured at each step. A priming dose of GKA23 (10 mg/kg) was given i.v. at 0 min followed by a constant infusion of GKA23 (5.5 mg/kg/hour) throughout the study. Glucose levels were clamped at step 1 (120 mg/dl), step 2 (180 mg/dl), and step 3 (240 mg/dl). (D) Glucose infusion rate (GIR) during the hyperglycemic step clamp. (E) Plasma C-peptide and (F) insulin concentration during hyperglycemic clamp. Statistical significance comparing vehicle and GKA23 is expressed as *P<0.05, **P<0.01, and ***P<0.001.

As a first step to assess whether GKA23 could improve glucose metabolism in vivo, we treated Sprague Dawley rats with vehicle or GKA23 (30 mg/kg) and performed an oral glucose tolerance test. Acute intraperitoneal (i.p.) administration of GKA23 reduced glucose levels in the fasting state (0 minute, Fig. 2A). Glucose levels following the exogenous glucose challenge were also lower than in vehicle-treated animals, but the area-under-the-curve (AUC) from 0 to 90 minutes was the same, indicating no change in glucose tolerance in this experiment (Fig. 2A and S1). Insulin levels after glucose challenge were comparable between the vehicle- and GKA23-treated groups (Fig. 2B), with a non-significant trend towards increased insulin immediately prior to the glucose challenge (Fig. 2B; 0 minute). To quantitate insulin secretion by GKA23 at various glucose levels, we performed a hyperglycemic step clamp study in rats. In this study, blood glucose levels were clamped at euglycemic (120 mg/dl) and hyperglycemic (180 and 240 mg/dl) levels (Fig. 2C). Based on modeling of single-dose i.p. and intravenous (i.v.) pharmacokinetic data (J. Davda, unpublished), we administered GKA23 as a bolus 10 mg/kg i.p. injection followed by a constant 5.5 mg/kg/hr i.v. infusion to target steady state drugs levels of 15 µM during the course of the study. Consistent with expectations, plasma GKA23 levels measured at the end of the study were 11.7 µM (data not shown). A markedly greater glucose infusion rate was required in the GKA23-treated rats (Fig. 2D), demonstrating improved glucose metabolism. Both vehicle- and GKA23-treated rats showed elevated C peptide and insulin levels following exogenous glucose infusion, indicating glucose-induced insulin secretion (Fig. 2E and 2F). Compared to vehicle treatment, the GKA23 infusion caused a trend towards higher C peptide and insulin levels (Fig. 2E and 2F), but it was not statistically significant. Because insulin secretion was only marginally increased in these experiments, we conclude that the greater glucose infusion rate in the GKA23-treated rats was primarily due to direct hepatic effects of the compound.

**Figure 3:**
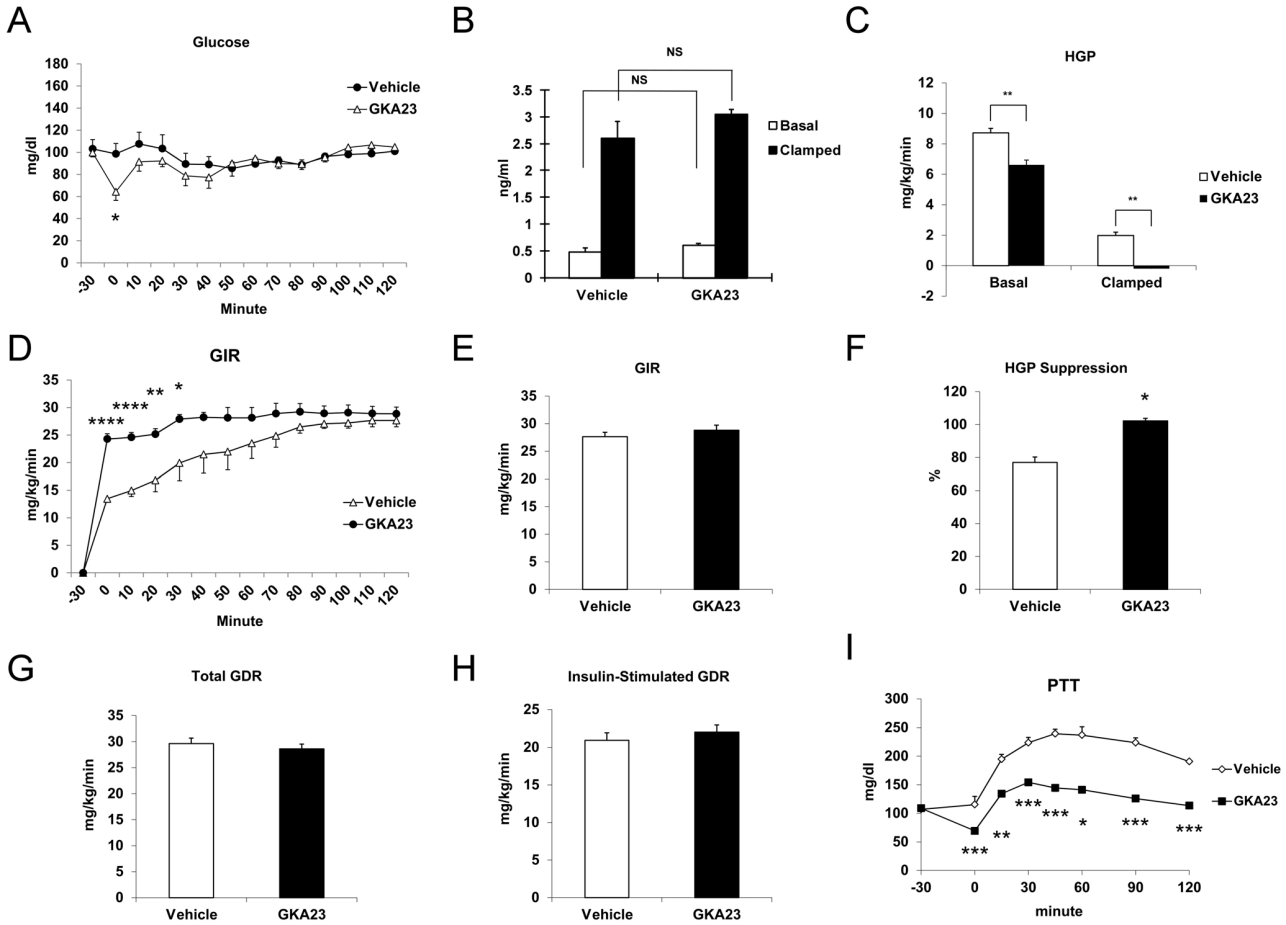
GKA23 decreases hepatic glucose production. (A) Glucose levels during the euglycemic hyperinsulinemic clamp study in control or GKA23 treated rats (n = 6/group). A bolus injection of GKA23 (30 mg/kg) was given i.p. at 30 min before insulin infusion. Glucose levels were clamped at 100 mg/dl. (B) Basal and clamped insulin levels in vehicle- and GKA23-treated mice. (C) Basal and clamped hepatic glucose production (HGP). (D) Glucose infusion rate (GIR) during the clamp study. (E) GIR at steady state in the final 20 minutes of the clamp study. (F) HGP suppression by insulin during the clamp study (G) Total glucose disposal rate (GDR). (H) Insulin-stimulated glucose disposal rate (IS-GDR) during the clamp study. (I) Glucose levels during the pyruvate tolerance test (PTT; 1 g/kg) on 6 hour-fasted rats (n = 6/group). Vehicle or GKA23 (30 mg/kg) was administered i.p. at −30 min and pyruvate was administered i.p. at 0 min. Statistical significance comparing vehicle and GKA23 is expressed as *P<0.05, **P<0.01, ***P<0.001, and ****P<0.0001. NS, not significant.

In order to further understand the mechanisms by which GKA23 could improve glucose homeostasis, we conducted a euglycemic-hyperinsulinemic clamp study in rats to quantitatively measure in vivo insulin sensitivity. Glucose values during the course of the experiment are shown in Figure 3A, and basal insulin values, which were not significantly different between vehicle and GKA23-treated groups (p = 0.1), are shown in Figure 3B. Basal rates of hepatic glucose production (HGP) were significantly reduced by GKA23 treatment (Fig. 3C), which, in conjunction with the lack of change in insulin, suggests a direct effect on the liver. During the clamp, GKA23-treated animals had a slight, non significant (p = 0.2), increase in insulin levels (Fig. 3B). Glucose infusion rate was similar between the two groups (Fig. 3D and 3E) but HGP was significantly decreased (Fig. 3C), and the percent suppression of HGP was increased (Fig. 3F). However, the absolute change in HGP before and during the clamp (∼6.8 mg/kg/min) was similar between the vehicle and GKA23-treated animals, indicating no change in hepatic insulin sensitivity. There was no significant change in total or insulin-stimulated glucose disposal rate (GDR or IS-GDR, respectively, Fig. 3G and 3H). Since 70–80% of IS-GDR is attributable to skeletal muscle [13], this implies that under hyperinsulinemic conditions, GKA23 does not modulate insulin action on skeletal muscle. In a separate study to further investigate the hepatic effects of GKA23, we performed a pyruvate tolerance test in rats fasted for 6 hours. Following pyruvate injection, vehicle-treated animals showed the expected increase in plasma glucose levels resulting from increased hepatic gluconeogenesis (Fig. 3I). In contrast, GKA23-treated animals showed significantly reduced glucose levels at all time points measured (Fig. 3I and S2), a finding consistent with the reduced HGP seen in Figure 3C.

**Figure 4:**
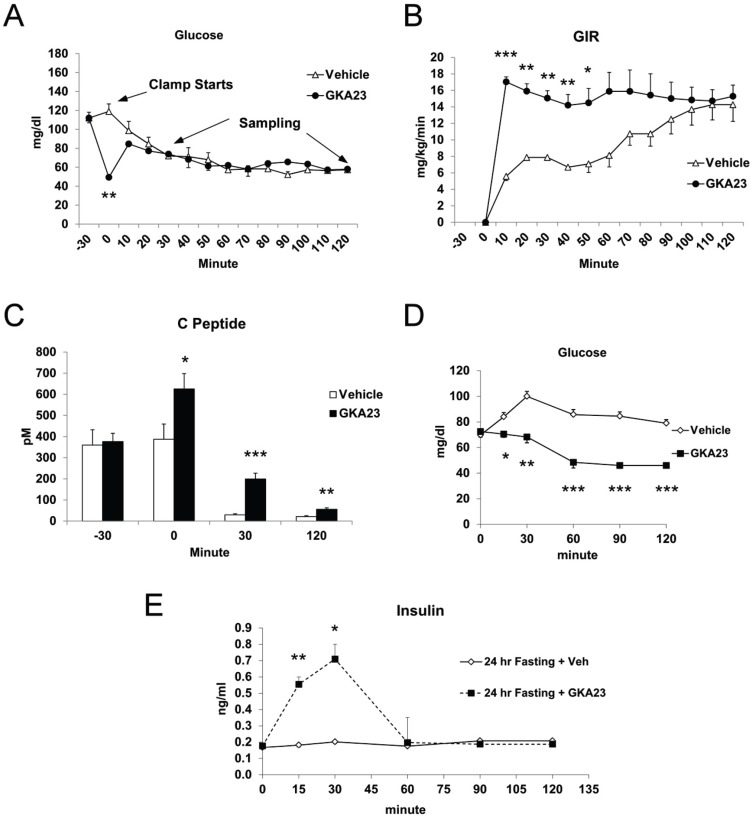
GKA23 induces insulin secretion in rats in the euglycemic and hypoglycemic state. (A) Hypoglycemic step clamp studies in control or GKA23-treated rats (n = 6/group). A bolus injection of GKA23 (30 mg/kg) was given i.p. at −30 min and the clamp study was initiated by insulin infusion at 0 min. Glucose levels were clamped at 60 mg/dl. (B) Glucose infusion rate (GIR) during the hypoglycemic clamp. (C) Plasma C peptide concentration during hypoglycemic clamp. (D) Blood glucose levels after vehicle or GKA23 injection (30 mg/kg) in 24 hour-fasted rats. (E) Plasma insulin concentration after vehicle or GKA23 injection in 24 hour-fasted rats. Statistical significance comparing vehicle and GKA23 is expressed as *P<0.05, **P<0.01, and ***P<0.001.

Hypoglycemia induced by insulin secretagogues is a common risk associated with this class of agents [3]. To examine whether GKA23 treatment induced persistent insulin secretion during hypoglycemia, we performed a hypoglycemic clamp study using low dose exogenous insulin levels infusion. As seen previously (Fig. 2A), treatment with GKA23 significantly reduced glucose levels (Fig. 4A; 0 minute), resulting in a greater glucose infusion rate required to maintain the target level of 60 mg/dl (Fig. 4B). Endogenous insulin levels, reflected by measured C peptide, were comparable at the beginning of the experiment but were significantly elevated following treatment with GKA23 (Fig. 4C; 0 minute). This elevation persisted through the end of the 2 hour time course, with the difference between the treated and untreated groups being larger at 30 and 120 minutes compared to 0 minutes. In a more physiological setting, we investigated the ability of GKA23 to induce insulin secretion in rats after prolonged fasting. Following a 24 hour fast, basal glucose levels were approximately 70 mg/dl (Fig. 4D). Administration of GKA23 significantly lowered glucose levels and increased insulin secretion compared to vehicle-treated animals (Fig. 4D and 4E). Therefore, in the settings of both insulin-and fasting-induced hypoglycemia, GKA23 can still effectively stimulate endogenous insulin secretion.

**Figure 5:**
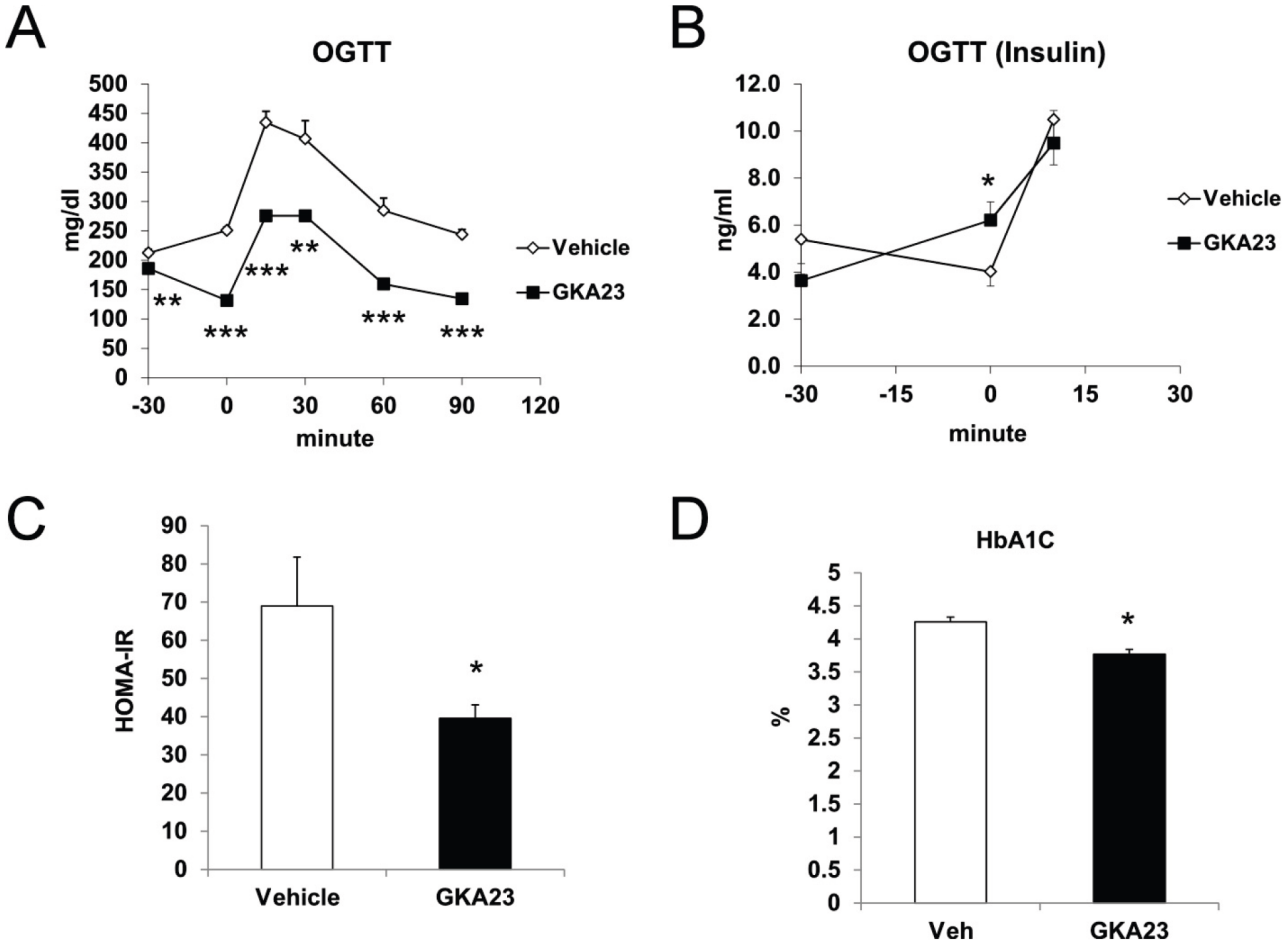
Sub-chronic GKA23 treatment improves glucose tolerance in mice fed a high-fat diet. (A) Oral glucose tolerance tests (OGTT; 1 g/kg) on 6 hour-fasted HFD mice (n = 8/group). Vehicle or GKA23 (30 mg/kg) was given by i.p. injection daily for 12 days. On the day of OGTT day 12, treatment was administered again at −30 min and glucose was orally gavaged at 0 min. (B) Plasma insulin concentration during OGTTs in 6 hour-fasted HFD mice. (C) HOMA-IR calculated from fasting plasma glucose and insulin levels in (A) and (B). (D) Blood hemoglobin A1C (HbA1C) in vehicle and GKA23-treated HFD mice (n = 8/group). Statistical significance comparing vehicle and GKA23 is expressed as *P<0.05, **P<0.01, and ***P<0.001.

**Figure 6:**
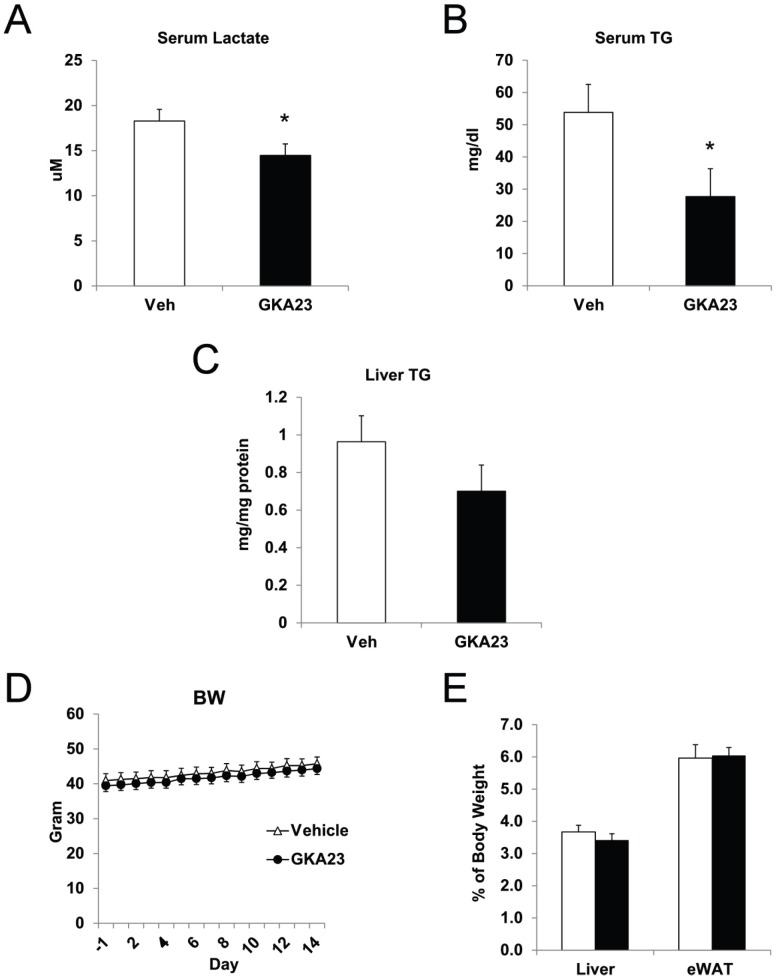
Sub-chronic GKA23 treatment improves glycemic control and lowers plasma triglyceride levels in mice fed a high-fat diet. (A) Serum lactate and (B) plasma triglyceride levels in vehicle and GKA23-treated HFD mice. (C) Hepatic triglyceride content in vehicle and GKA23-treated HFD mice. (D) Body weights as measured over the course of the study. (E) Liver and adipose tissue weights at the end of the study, expressed as a percent of body weight. Statistical significance comparing vehicle and GKA23 is expressed as *P<0.05, **P<0.01, and ***P<0.001.

We next evaluated the efficacy of GKA23 following sub-chronic treatment of obese, insulin resistant mice. Mice were fed a high fat diet for 12 weeks, treated for 12 days with GKA23 (30 mg/kg i.p., once daily), and then subjected to an oral glucose tolerance test. GKA23 treatment significantly reduced basal (6 hour-fasted) glucose levels (measured 24 hours after the previous GKA23 treatment on day 11) with a non-significant decrease in basal insulin levels (Fig. 5A and 5B; −30 minute time point). Calculation of HOMA-IR using fasted glucose and insulin values demonstrated a significant improvement in insulin sensitivity (Fig. 5C). Following GKA23 treatment but before the glucose challenge (Fig. 5A and 5B, 0 minute time point), glucose levels were further reduced and insulin significantly increased. Post-challenge glucose was significantly reduced for all time points, demonstrating improved glucose tolerance. Because the GKA23-treated mice had comparable insulin levels at lower glucose levels (Fig. 5A and 5B, 10 minute time point), this also suggests improved insulin secretion. Long-term glycemic control was improved by GKA23 treatment, as indicated by the significantly lower HbA_1c_ levels measured after 14 days (Fig. 5D). A concern with GK activation in the liver is that increased intracellular glucose flux could lead to elevated hepatic triglycerides (TGs), serum TGs, and/or serum lactate levels [14]. To investigate these possibilities, we also measured these parameters at day 14. Serum TG and lactate concentrations were significantly lower in the mice treated with GKA23 (Fig. 6A and 6B). Hepatic TG levels were slightly lower, but the change did not reach statistical significance (Fig. 6C). Body, liver and adipose tissue weights were unchanged (Fig. 6D and 6E).

## Discussion

The essential role that GK plays in maintaining glucose homeostasis makes it a potentially attractive drug target for the treatment of type 2 diabetes. In the current study, we have examined the efficacy and mechanism of action of a new GKA in rats and mice. Overall, our data are consistent with the known biology of GK and with previous reports characterizing other GKAs [15], [16], [17], [18]. GKA23 improved glucose tolerance by stimulating insulin secretion, reducing hepatic glucose output, and increasing hepatic insulin sensitivity. These findings add additional support to the idea that GKAs could be important therapeutics for patients with type 2 diabetes.

Since GK is expressed in both liver and pancreatic islets, the glucose lowering effect of GK activation could result from the enhancement of insulin secretion, suppression of hepatic glucose production, or both. Our data show that GKA23 regulates both pathways in glucose metabolism. Interestingly, the insulin secretagogue effect of GKA23 seems dependent on blood glucose levels. An increase of insulin was seen in both euglycemic and hypoglycemic conditions, but only a very modest effect was seen during the hyperglycemic clamp study, despite a large potential window based on the insulin levels at the different clamp steps (Fig. 2E and 2F). GKA23 significantly elevated GIR at all steps during the hyperglycemic clamp study. GIR reflects whole body glucose clearance [13], but because GKA23 had no effect on muscle or other peripheral tissues (Fig. 3) the increase is most likely due to hepatic activity. Additional support for hepatic effects of GKA23 comes from the reduced glucose excursion following the pyruvate challenge (Fig. 3I) and the increased glucose uptake seen in primary rat hepatocytes (Fig. 1C).

Despite the beneficial effects on GKAs on multiple metabolic parameters, significant concerns remain about the overall safety of these agents. For example, the propensity of GKAs to cause hypoglycemia has been noted in clinical trials and is of obvious concern [11]. We investigated this aspect of GK biology using a hypoglycemic clamp and in rats fasted for 24 hours. Under these conditions, GKA23 demonstrated robust efficacy in both lowering glucose (in the hypoglycemic clamp this manifested as increased glucose infusion rate) and increasing insulin secretion. Furthermore, in rats fasted for 6 hours, GKA23 inhibited glucose production during a pyruvate tolerance test. These data indicate that there is no protective mechanism to prevent hypoglycemia induced by GKA23 treatment.

The potential for elevated triglycerides is another long-standing concern for GKAs [11], [19]. Rats over-expressing hepatic GK have elevated TGs [14], and humans studies have shown both that increased expression of hepatic GK is associated with increased hepatic lipogenic gene expression and TG content [20], and that polymorhpisms in the gene encoding the glucokinase regulatory protein (GKRP), a negative regulator of glucokinase, increase plasma TGs [21]. However, patients with activating mutations in GK do not always show abnormal blood lipids [22], [23], [24]. Pharmacologically, the picture is also mixed. One recent report found significantly elevated hepatic TGs following treatment of db/db mice and Wistar and ZDF rats with several GKAs [19]. However, a different group showed that a different GKA did not increase hepatic TGs in ZDF rats [10], and yet another group published that while one GKA and a structurally-related but inactive analogue caused hepatic lipidosis in Wistar rats, other GKAs did not [9]. Taken together, and despite a plausible mechanistic connection between GK activation and hepatic lipid accumulation, these data suggest the findings may be off-target. Our data are consistent with this idea, as we did not see any increase in hepatic triglycerides following two week treatment of mice fed a high fat diet, and in fact plasma triglycerides were significantly reduced. How these findings would relate to patients with type 2 diabetes is unclear. In clinical trials with the GKA MK-0941, elevated plasma TGs were seen in some studies but not others [11].

GKA23 treatment effectively improved fasting and post-OGTT glucose in obese, insulin resistant mice (Figs. 5 and 6). The response was durable for 2 weeks, improved HOMA-IR, and reduced HbA1c. These results are generally consistent with the preclinical results reported for other GKAs, and with data from some, but not all, studies with transgenic or viral over-expression of GK [25], [26]. Clinically, a lack of durable response was seen in both the MK-0941 and AZD1656 Phase 2 trials, where initial reductions in HbA_1c_ were not maintained through the end of the studies [11], [12]. By comparison, our preclinical study may not have been long enough for a potential lack of sustained efficacy to become apparent, or our result may not be predictive of what could be observed in humans. Additional studies will be required to explore this and other characteristics of GKA23.

## Supporting Information

Figure S1 Plasma glucose AUC from the OGTT shown in Figure 2A. AUC from 0 to 90 minutes from the glucose tolerance test shown in Figure 2A. For each group, the value at t = 0 was subtracted from the other time points.
(TIF)

Figure S2 **P**lasma glucose AUC from the pyruvate tolerance test. AUC from 0 to 120 minutes from the pyruvate tolerance test shown in Fig. 3I. For each group, the value at t = 0 was subtracted from the other time points. Statistical significance comparing vehicle and GKA23 is expressed as **P<0.01.
(TIF)
