# Supplementary figures and images for: Characterization of a Novel Glucokinase Activator in Rat and Mouse Models
Source: PLoS One. 2014 Feb 12;9(2):e88431. doi: 10.1371/journal.pone.0088431 (PMC3922816; doi:10.1371/journal.pone.0088431)

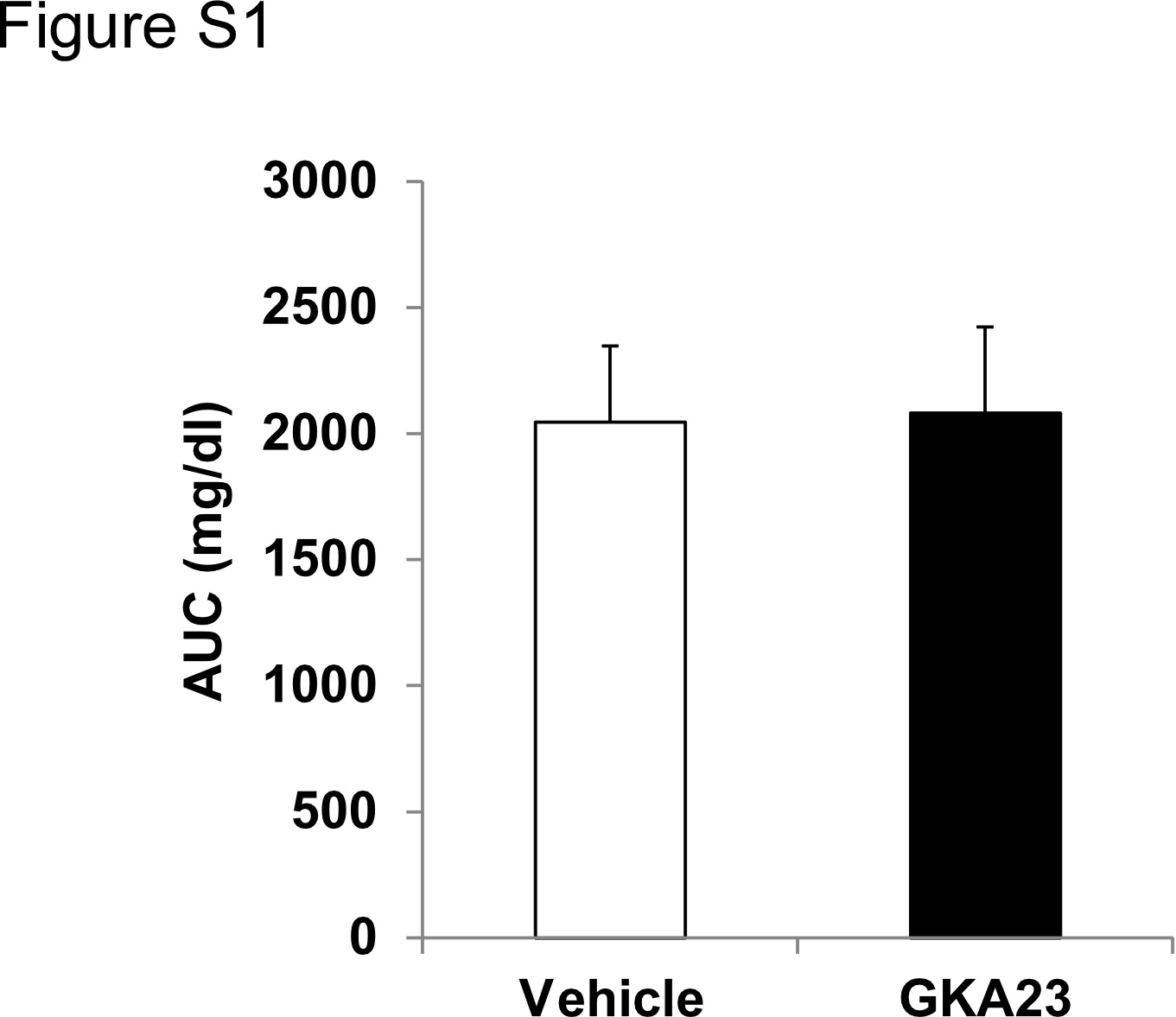

Supplement: Figure S1 — Plasma glucose AUC from the OGTT shown in Figure 2A. AUC from 0 to 90 minutes from the glucose tolerance test shown in Figure 2A. For each group, the value at t = 0 was subtracted from the other time points. (TIF) [file pone.0088431.s001.tif]

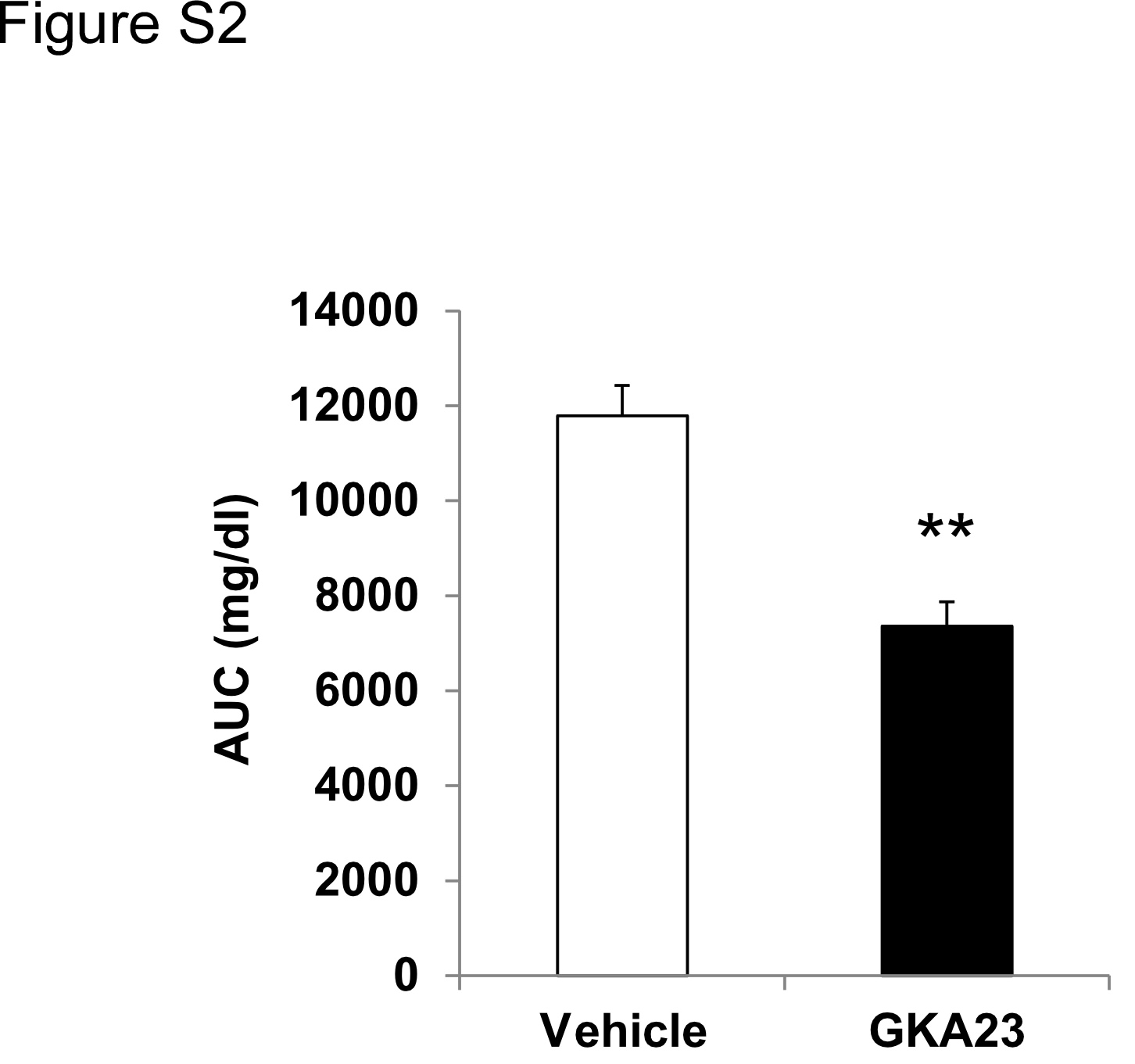

Supplement: Figure S2 — Plasma glucose AUC from the pyruvate tolerance test. AUC from 0 to 120 minutes from the pyruvate tolerance test shown in Fig. 3I. For each group, the value at t = 0 was subtracted from the other time points. Statistical significance comparing vehicle and GKA23 is expressed as **P<0.01. (TIF) [file pone.0088431.s002.tif]
